# Adaptations of *Escherichia coli* strains to oxidative stress are reflected in properties of their structural proteomes

**DOI:** 10.1186/s12859-020-3505-y

**Published:** 2020-04-29

**Authors:** Nathan Mih, Jonathan M. Monk, Xin Fang, Edward Catoiu, David Heckmann, Laurence Yang, Bernhard O. Palsson

**Affiliations:** 1 Department of Bioengineering, University of California San Diego, La Jolla, CA 92093 USA; 2 Bioinformatics and Systems Biology Program, University of California San Diego, La Jolla, CA 92093 USA; 3 The Novo Nordisk Foundation Center for Biosustainability, Technical University of Denmark, DK-2800 Kgs, Lyngby, Denmark

**Keywords:** Structural systems biology, Oxidative stress, Structural proteome, Physicochemical properties, Oxidative damage, Metabolic model

## Abstract

**Background:**

The reconstruction of metabolic networks and the three-dimensional coverage of protein structures have reached the genome-scale in the widely studied *Escherichia coli* K-12 MG1655 strain. The combination of the two leads to the formation of a structural systems biology framework, which we have used to analyze differences between the reactive oxygen species (ROS) sensitivity of the proteomes of sequenced strains of *E. coli*. As proteins are one of the main targets of oxidative damage, understanding how the genetic changes of different strains of a species relates to its oxidative environment can reveal hypotheses as to why these variations arise and suggest directions of future experimental work.

**Results:**

Creating a reference structural proteome for *E. coli* allows us to comprehensively map genetic changes in 1764 different strains to their locations on 4118 3D protein structures. We use metabolic modeling to predict basal ROS production levels (ROStype) for 695 of these strains, finding that strains with both higher and lower basal levels tend to enrich their proteomes with antioxidative properties, and speculate as to why that is. We computationally assess a strain’s sensitivity to an oxidative environment, based on known chemical mechanisms of oxidative damage to protein groups, defined by their localization and functionality. Two general groups - metalloproteins and periplasmic proteins - show enrichment of their antioxidative properties between the 695 strains with a predicted ROStype as well as 116 strains with an assigned pathotype. Specifically, proteins that a) utilize a molybdenum ion as a cofactor and b) are involved in the biogenesis of fimbriae show intriguing protective properties to resist oxidative damage. Overall, these findings indicate that a strain’s sensitivity to oxidative damage can be elucidated from the structural proteome, though future experimental work is needed to validate our model assumptions and findings.

**Conclusion:**

We thus demonstrate that structural systems biology enables a proteome-wide, computational assessment of changes to atomic-level physicochemical properties and of oxidative damage mechanisms for multiple strains in a species. This integrative approach opens new avenues to study adaptation to a particular environment based on physiological properties predicted from sequence alone.

## Background

Reactive oxygen species (ROS) can cause severe oxidative damage to cellular proteins, which often results in chain reactions that spread the damage to neighboring macromolecules and leads to systems-level changes of cellular function [1]. While ROS can be beneficial - and even necessary in some contexts [2–5] - at a high concentration, oxidative damage must be responded to and repaired. As a result, cells are equipped with a number of mechanisms to quench ROS, directly repair the damaged components, or manage ROS indirectly [6]. Certain amino acids that constitute the structures of proteins are principal sites of oxidative damage [7]. This damage can be divided into two groups - reversible or irreversible modifications. The sulfur-containing residues methionine and cysteine incur reversible modifications, while irreversible damage impacts histidine, arginine, lysine, proline, threonine (RKPT), and tyrosine (with reactive nitrogen species) [8]. The damage to the RKPT amino acids is a post-translational modification known as carbonylation, and is commonly used as an experimental measurement of oxidative damage with mass spectrometric methods [9–11].

A number of studies have explored the adaptations of an organism’s proteome to deal with an oxidative environment. These studies have examined how the amino acid usage of their proteomes differs between anaerobes versus aerobes [12], or between short- and long-living organisms [13–15]. The latter case has been of interest due to the proposed oxidative stress theory of aging [16] which states that the accumulation of oxidative damage to macromolecules is a major reason why organisms age. As a result of these studies, there are a number of proposed hypotheses regarding how aerobic organisms have evolved to live in oxygen-rich environments or why certain species have increased longevity. In the context of the structural proteome, these proposals include: 1) the use of “amino acid sponges” that can absorb oxidative damage by enriching cytosolic protein surfaces with methionine [17–20] or cysteine [21], and additionally tyrosine or tryptophan in transmembrane proteins [22]; 2) the avoidance of cysteines [23] or carbonylation-susceptible residues [10, 24] on the surface of a protein; 3) the avoidance of charged or disorder-prone residues [25–28] to favor a more stable folded state; 4) the protection of reactive cofactors such as transition metal ions or flavins with extended domains [29] or by altering global or local structural characteristics [30–32]; and 5) a number of other novel mechanisms [33–37]. The true impact of these adaptations remains unclear as patterns observed could be attributed to other factors [12], but it is clear from these studies that antioxidative protein properties have manifested themselves within the genetic code over time.

**Fig. 1:**
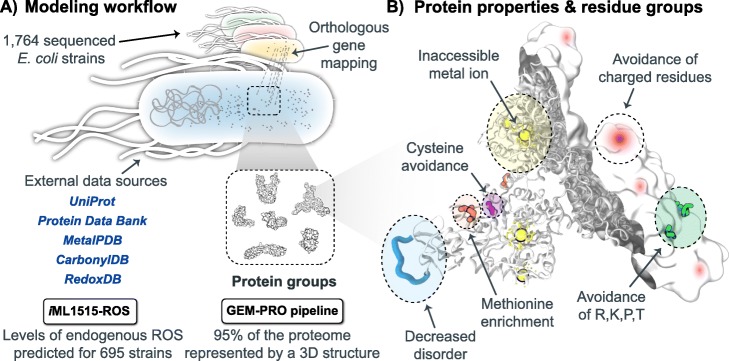
Schematic of modeling workflow and the hypothetical antioxidative properties of a protein. **a** The genomes of 1764 strains of *E. coli* were gathered and orthologous genes were mapped to the reference *E. coli* K-12 MG1655 strain. External data sources were integrated to gather protein sequence and structure annotations with regards to susceptibility of oxidative damage, such as the locations of metal-binding sites [38], known carbonylation sites [39], and known cysteine damage sites [40]. The structural proteome is further categorized into protein groups by their annotated localization and functionality (see Additional file 1: Table S1). We conducted gene deletions upon the genome-scale metabolic model of MG1655 integrated with ROS generating reactions (*i*ML1515-ROS [41, 42]) for strain-specific predictions of basal ROS production levels, defining a strain’s “ROStype”. We utilized the GEM-PRO pipeline [43, 44] to select representative protein structures for 95% of the MG1655 proteome. **b** A hypothetical protein resistant to oxidative damage. Protein sequence and structure properties are highlighted based on previous studies finding enrichment of these properties in aerobes or long-living organisms. Structural properties defined by locations in 3D space, such as surface-exposed residues or those in a specified radius within a metal-binding site, are used to further divide a single protein into residue groups (see Additional file 1: Table S3)

In this study, we use a combination of both structural and systems biology approaches to evaluate if the differential manifestation of these antioxidative properties in the proteomes of multiple *E. coli* strains reflects the varying oxidative environments they may encounter. We extend a pipeline to construct genome-scale models with protein structures (GEM-PROs) to the entire reference proteome of *E. coli* K-12 MG1655, with additional methods to select representative cofactor-bound structures and protein complexes. We use this information along with a set of 1764 sequenced *E. coli* strains to map DNA sequence variation to the reference proteome, with the goal of characterizing physicochemical changes in groups of proteins defined by their localization and functionality (Fig. 1). We additionally create strain-specific genome-scale metabolic models capable of predicting basal ROS production levels (henceforth referred to as “ROStypes”) [41, 42] to understand how adaptation may arise not only due to levels of ROS encountered exogenously, but also produced endogenously. With this information, we are able to pinpoint shared changes in relation to a strain’s phenotype and the antioxidative properties of its proteome. We unexpectedly find that strains with predicted higher levels of endogenous ROS relative to MG1655 (ROS^hi^) share antioxidative properties in their proteomes to those with predicted lower levels (ROS^lo^). We find that generally, metalloproteins and periplasmic proteins differ in these antioxidative properties, and detail two specific examples of molybdenum-binding enzymes and proteins involved in the biogenesis of fimbriae. This work demonstrates a structural systems biology approach to explore patterns of protein sequence variation in relation to predicted or known phenotypes, specifically in the context of adaptation to an oxidative environment.

## Results

### Reconstructing the reference structural proteome of *E. coli* K-12 MG1655

The construction of a reference structural proteome for the 4313 proteins of the *E. coli* K-12 MG1655 strain resulted in 1457 proteins that could be represented by an experimentally determined 3D structure, an additional 2661 proteins with a homology model, and 195 with no available structure. Proteins were segregated into functionally similar groups, first based on their localization within the cell and further using clusters of orthologous group (COG) categories and metabolic subsystem groupings, resulting in over 200 groups of proteins (henceforth referred to as *protein groups*) analyzed for differences in their structural properties that could potentially contribute to oxidative stress resistance (henceforth referred to as *antioxidative properties*) (Fig. 1) (see Additional file 1: Table S1). These antioxidative properties were selected on the basis of previous studies that characterized hypothesized resistance patterns seen in aerobes or in long-living organisms, and are listed in Additional file 1: Table S3.

Improvements to the GEM-PRO pipeline [43, 44] allowed for the refined selection of experimental protein structures for 42 iron and iron-sulfur binding enzymes. For these, selection of a representative structure was extended beyond sequence identity and structural resolution by considering cofactor-bound states if experimental structures were available in both apo (cofactor-unbound) and holo (cofactor-bound) forms. The integration of external data sources enabled the assessment of changes to experimentally determined damage points on proteins for carbonylation (24 proteins with 84 total experimentally carbonylated residues) and cysteine damage (94 proteins with 150 total experimentally damaged cysteines). These improvements result in a more rigorous reconstruction of the structural proteome and also enable future analyses to consider important protein subsequences (Fig. 1b), such as for changes within a certain radius of a metal-binding site or known sites of damage.

### Strains with significant variance in ROStypes display enrichment of antioxidative properties in their proteomes

**Fig. 2:**
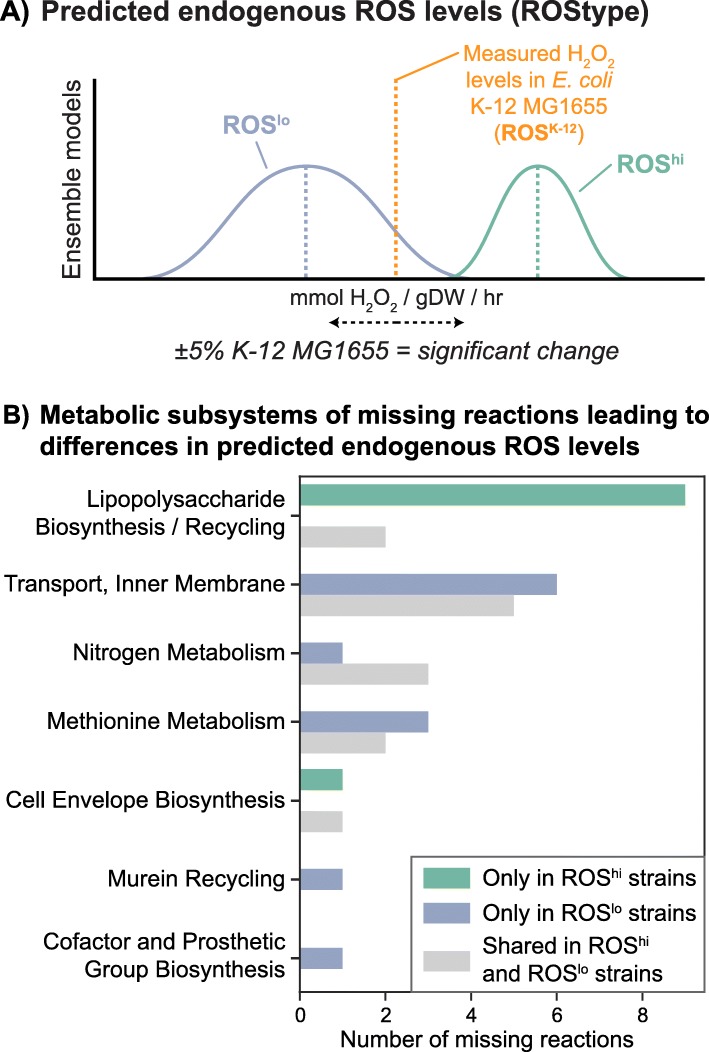
Classifying strains by predicted endogenous ROS levels and missing reactions that contribute to the predicted phenotype. **a** Simulations of strain-specific metabolic models enable the prediction of endogenous ROS levels, or ROStype. A defined ROStype results from changes to pathway usage due to the deletion of certain reactions from missing genes. Strains are classified by their predicted endogenous ROS levels as “high” (ROS^hi^) or “low” (ROS^lo^) ROStypes if their predicted rates of production of hydrogen peroxide (H_2_O_2_) or superoxide (O_2_^−^) differ by more than 5% from the measured production rate in the K-12 MG1655 strain (orange dotted line). If predicted endogenous ROS levels do not differ by more than 5%, a strain is classified as similar to MG1655 (ROS^K-12^). **b** Histogram of the metabolic subsystems of missing reactions that contribute to the ROStype. Reactions that are shared between strains with ROS^hi^ and ROS^lo^ predictions are denoted as shared missing reactions in gray

The protein sequences of 1764 strains of *E. coli* were gathered from public databases (see Methods). Simulations to predict the ROStype of available *E. coli* strains were successful for 695 strains, and resulted in a set of 16 strains that had a significantly higher basal ROS production rate (ROS^hi^, > 105% measured K-12 MG1655 production rate [45]) and 26 that had a lower basal rate (ROS^lo^, < 95% measured rate) (Fig. 2a). This cutoff was selected from a previous study that validated a number of gene deletions and their impact on measured endogenous ROS levels [41]. The production rate of H_2_O_2_ and O_2_^−^ was highly correlated (*r* = 0.98) and thus, classification of a strain based on their ROStype refers to production rates of both H_2_O_2_ and O_2_^−^. A large number of strains (653) displayed non-varying levels (within ±5%) compared to K-12 MG1655 (ROS^K-12^) while the remaining strains (1069) failed to simulate growth under minimal media conditions, most often due to missing amino acid synthesis pathways that were not mapped from orthologous genes. Gap-filling of the strain-specific metabolic models was not carried out.

**Fig. 3:**
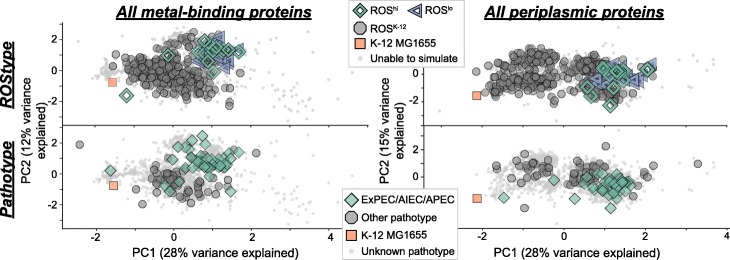
Principal components analysis (PCA) of antioxidative properties in all metal-binding proteins as well as all periplasmic proteins. Antioxidative properties are computed for all metal-binding proteins and averaged for every strain. A feature matrix containing these properties is used as input to PCA, and subsequently, strains with predicted ROStypes (top left) and strains with pathotype annotations (bottom left) are highlighted. PCA for all proteins localized to the periplasm is also shown (right). These two general groups of proteins show clusters with the highest homogeneity in regards to predicted endogenous ROS or pathotype labels

**Fig. 4:**
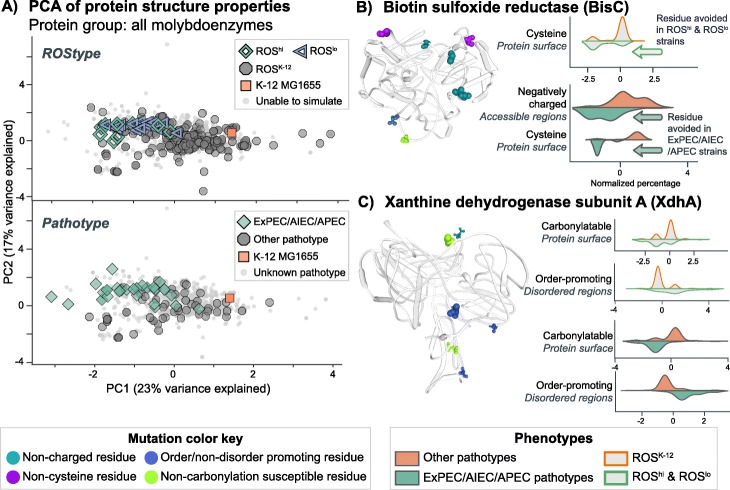
Molybdenum-binding proteins are enriched in antioxidative properties in strains with both high and low predicted levels of endogenous ROS as well as strain pathotypes likely encountering oxidative environments. **a** PCA of antioxidative properties for molybdenum-binding proteins of strains, in relation to their predicted ROStype and annotated pathotype. Proteins were inspected individually for changes in antioxidative properties, since analysis of the component contributions showed both enrichment and avoidance of certain properties. **b** Biotin sulfoxide reductase shows avoidance of surface-exposed cysteine residues in both ROS^hi^/ROS^lo^ and ExPEC/AIEC/APEC strains. The residues highlighted on the protein structure indicate common mutations in strains of ExPEC/AIEC/APEC pathotypes. The size of the highlighted residue corresponds to the number of strains that mutation appears in. Note that all mutations do not co-occur together in all strains. The distribution plots for strain phenotypes to the right of the protein figure show the normalized percentage of the residue in relation to the protein subsequence, i.e. percentage of cysteines on the protein surface. **c** Xanthine dehydrogenase subunit A similarly shows enrichment of antioxidative properties, by avoiding carbonylatable and charged residues on the protein surface, along with an increase of a total percentage of order-promoting residues. Structural models shown here are all homology models from the SWISS-MODEL database [48]

**Fig. 5:**
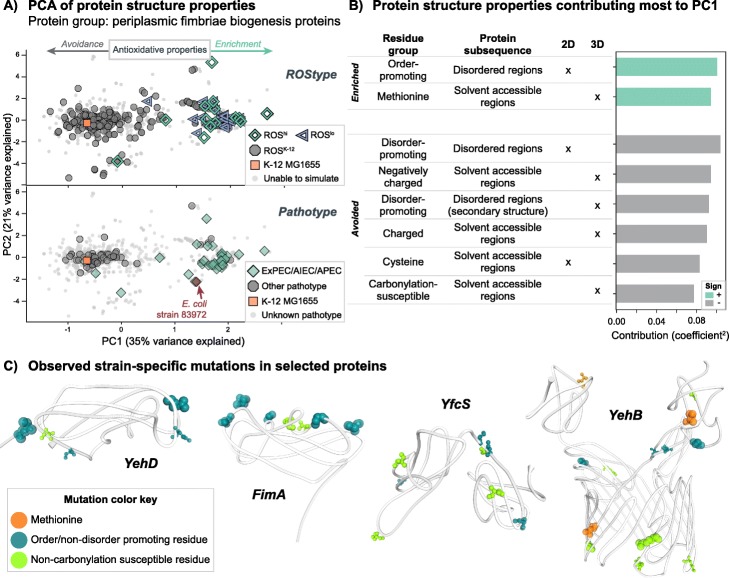
Proteins in the periplasm involved in the assembly of fimbriae are enriched in antioxidative properties. **a** PCA of antioxidative properties for periplasmic fimbriae assembly proteins, in relation to their simulated ROStype and annotated pathotype. PC1 separates the observed ExPEC/AIEC/APEC strains well, confirmed by DBSCAN clustering (Table 1, Table 2). The location of two asymptomatic strains (83972) is specified as they have been found to outcompete UPEC strains in adhesion of the bladder wall [51]. **b** Specific antioxidative properties contributing to PC1. The 2D and 3D columns indicate if the protein subsequences were determined by predictions from sequence (2D) or calculations from structure (3D). **c** Selected examples from the proteins involved in fimbriae assembly that show enrichment of antioxidative properties. YehB, YehD, and YfcS are relatively unknown components of operons similar to the *fim* operon [52]. Up to 3000 copies of FimA form the structural pilus of the fimbriae [53]. Highlighted residues indicate mutations which are seen in the cluster with the most ExPEC/AIEC/APEC pathotypes. The size of the highlighted residue corresponds to the number of strains that mutation appears in. Note that all mutations do not co-occur together in all strains. The structures shown here are from a collection of homology models from the SWISS-MODEL and I-TASSER modeling pipelines [48, 54]

From the set of ROS^hi^ and ROS^lo^ strains, we inspected the gene deletions that resulted in the predicted phenotypes. Computationally, this could have been the case due to a common missing reaction from a set of sequenced strains that had a shared quirk, due to sequencing errors or other technical problems. Closer inspection of the missing reactions revealed no defined set of gene deletions that lead to a ROS^hi^ or ROS^lo^ phenotype, however, there are similarities between the two in terms of which reactions were eliminated. In both classes, the major subsystem of reactions missing due to gene deletions were involved in alternate carbon metabolism and lipopolysaccharide biosynthesis, consistent with other studies of the core and pan genome [46, 47]. Both strain sets shared missing reactions in methionine metabolism, nitrogen metabolism, and inner membrane transport pathways. ROS^hi^ strains were missing additional lipopolysaccharide (LPS) biosynthesis reactions, and the only non-LPS reaction unique to these strains was a deletion of UDP-galactopyranose mutase. ROS^lo^ strains on the other hand, had a variety of missing reactions in different subsystems (Fig. 2a). Interestingly, in most protein groups, the computed antioxidative properties did not significantly cluster apart the ROS^hi^ and ROS^lo^ strains in principal component analysis (PCA) (top panels of Fig. 3a, Fig. 4a, Fig. 5a). It was observed that these strains clustered together, but apart from strains with non-varying levels of endogenous ROS. To verify that these antioxidative features were indeed unable to be used to cluster ROS^hi^ and ROS^lo^ strains apart from each other, a random forest classifier was trained and features were tuned using leave-one-out cross validation. The classifier performed poorly (AUC < 0.6) using these features in the reported protein groups of this work, except for the metal-binding enzymes which had a modest AUC of 0.86. This indicates that there may indeed be some discriminating features in the metal-binding enzymes of ROS^hi^ versus ROS^lo^ strains, which is not unexpected as they are main targets of damage.

### Known chemical mechanisms of oxidative damage to proteins enable the assessment of a strain’s oxidative environment

Pathotype classifications were available for a subset of 116 from the overall set of 1764 strains, while growth/no growth phenotypes in a variety of media conditions were available for up to 650 strains depending on the condition [55]. PCA of the selected antioxidative properties successfully segregated strains with predicted ROStypes (i.e., ROS^hi^ and ROS^lo^ strains vs. ROS^K-12^) and those with defined pathotypes (i.e., ExPEC/AIEC/APEC vs. other pathotypes) for a number of protein groups (Table 1, Table 2). These groups were ranked by cluster homogeneity following Density-Based Spatial Clustering of Applications with Noise (DBSCAN). Examples of proteins showing significant cluster homogeneity include the general protein groups of all metal-binding enzymes as well as all proteins localized to the periplasm (Fig. 3). Specifically, in these groups, the metal-binding enzymes that utilize a molybdenum cofactor and periplasmic enzymes involved in the assembly of fimbriae showed clear clustering. These specific groups are expanded upon below. Other protein groups with high homogeneity included other extracellular and motility proteins, murein biosynthesis enzymes, and proteins involved in lipid or inorganic ion transport and metabolism (Table 1, Table 2).

**Table 1:** Homogenous clusters formed using DBSCAN on the first two principal components, following labeling with ROStype

Protein group	Protein count	Cluster count	V-measure
COG I (Lipid transport and metabolism)	97	2	0.33
Metabolism - Murein recycling	40	3	0.30
Manganese-binding enzymes	33	2	0.29
Fimbriae assembly proteins	39	4	0.29
Metabolism - All metabolic proteins	1515	2	0.27
All metal-binding proteins	590	3	0.27
COG P (Inorganic ion transport and metabolism)	198	2	0.27
Metabolism - Inner membrane transport	299	2	0.26
All periplasmic proteins	315	3	0.14
Molybdenum-binding enzymes	14	4	0.14

For ROStypes, both high and low predictions were considered the same label in homogeneity measurements. If a ROStype is unavailable for a strain, it was excluded from the homogeneity measurements. A V-measure is the harmonic mean of clustering homogeneity and completeness measures [49].

**Table 2:** Homogenous clusters formed using DBSCAN on the first two principal components, following labeling with pathotype

Protein group	Protein count	Cluster count	V-measure
Fimbriae assembly proteins	24	2	0.47
Metabolism - All metabolic proteins	1515	2	0.44
COG Q (Secondary metabolites biosynthesis)	41	4	0.44
COG N (Cell motility)	27	3	0.41
Metabolism - Alternate carbon metabolism	231	3	0.39
Iron-sulfur-binding enzymes	118	2	0.38
Zinc-binding enzymes	125	3	0.37
All periplasmic proteins	315	2	0.37
All metal-binding proteins	590	3	0.33
Molybdenum-binding enzymes	14	2	0.31

For pathotypes, ExPEC/AIEC/APEC strains are grouped together as they likely encounter oxidative environments more frequently [50]. If a pathotype is unavailable for a strain, it was excluded from the homogeneity measurements.

We observed very little segregation of the panel of strains with available growth/no growth phenotypes in various media conditions [55], with our original hypothesis being that strains with growth phenotypes in oxidative environments would show enrichment of antioxidative properties in proteins important for metabolic function. A major reason for this observation was due to a much lower statistical power as many conditions contained a very small number of strains with “no growth” phenotypes compared to those with a “growth” phenotype.

### Molybdoenzymes with promiscuous activity are enriched in antioxidative properties

Enzymes that utilize molybdenum as an inorganic cofactor in catalysis carry out a variety of redox reactions in *E. coli* [56] and in all eukaryotic organisms [57, 58]. The molybdoenzyme group created through the structural proteome reconstruction was composed of 14 enzymes containing molybdenum-binding sites in their associated UniProt entries (see Additional file 1: Table S5). PCA of their antioxidative properties displayed clear clustering of ROStypes and pathotypes (Fig. 4a). However, analysis of the antioxidative properties contributing to the first principal component revealed conflicting properties among the proteins contained within the group (see XdhD, below). Thus, we proceeded to inspect the properties of each of the 14 molybdoenzymes individually.

The subset of these enzymes that show enrichment of antioxidative properties in the pathotype and endogenous ROS clusters display dual functionalities in many cases. For example, the main function of biotin sulfoxide reductase (BisC) is in biotin salvage (i.e., to reduce the oxidized form of biotin), but it has also been shown to reduce oxidized free methionine [59]. BisC most significantly shows an avoidance of surface-exposed cysteines and negatively-charged residues (*p* < 0.0001, Mann-Whitney U test) (Fig. 4b). An avoidance of surface-exposed methionines was also observed. Trimethylamine-N-oxide (TMAO) reductase 2 (TorZ) reduces the compound TMAO, an alternative electron acceptor for anaerobic growth [60, 61]. Additionally, TorZ carries out the same biotin sulfoxide reductase reaction as BisC [62], although at a lower catalytic rate. TorZ displayed properties in the same strain sets trending towards more ordered residues, and less surface-exposed carbonylatable and charged residues. Xanthine dehydrogenase subunit A (XdhA), which showed similar property enrichments as TorZ (Fig. 4c), is involved in purine catabolism and reduces NAD+ to NADH in the process [63]. Increases in xanthine levels are potentially indicative of higher rates of DNA damage, such as from ROS [64]. Out of the set of molybdoenzymes which avoided antioxidative properties, most are either known to only have a single function, or do not have their functions well characterized as of yet. As an example, a hypothesized xanthine oxidase (XdhD), displayed changes that shifted it to be more susceptible to damage, such as an enrichment of disordered and carbonylatable residues. These changes may be due to the fact that XdhD cannot carry out the dehydrogenase reaction that XdhA does, since it lacks the FAD-binding domain to catalyze it [63]. As such, being an oxidase, XdhD is involved in the generation of ROS and may not be desirable for use in high ROS environments.

### Operons containing type 1 fimbriae biogenesis proteins are enriched in antioxidative properties

Fimbriae are special pili that are synthesized and transferred to the outer membrane of *E. coli*. They are involved in the attachment of the bacteria to their host environments [65]. The *fim* operon is the most well characterized system that assembles type 1 fimbriae [66]. A number of other similar operons are encoded within the K-12 MG1655 genome, but require specific environmental stimuli to be expressed [52]. PCA and subsequent DBSCAN clustering identified this set of proteins as creating highly homogenous clusters, again for both strains with predicted ROStypes and defined pathotypes (Fig. 5a).

One cluster contained a majority of the annotated ExPEC/AIEC/APEC strains (36 of 38), along with two asymptomatic (ABU) 83,972 strains and 8 strains with a variety of other pathotypes (see Additional file 1: Table S6). Another cluster was comprised by a majority of EHEC strains (37 of 65). The antioxidative properties contributing to the first principal component were largely consistent with many of the previously outlined properties hypothesized to contribute to oxidative stress resistance. Specifically, the rightmost strains on PC1 are enriched in order-promoting residues in disordered regions of their proteins, along with solvent accessible methionines. The leftmost strains on PC1 are characterized by amino acid features opposite to providing oxidative stress resistance, such as an increase in disorder-promoting residues in disordered regions, more solvent accessible cysteines, charged residues, and residues susceptible to irreversible carbonylation (Fig. 5b). The proteins that diverged in sequence the most from the orthologous K-12 sequence were those in the *yeh* and *yfc* operons (Fig. 5c). The function of these operons is relatively unknown but hypothesized to assemble other type 1 fimbriae due to their homology to *fim* components [52].

## Discussion

### Endogenous ROS levels suggest convergent evolution in oxidative stress resistance

The principal component analyses of the antioxidative properties of protein groups revealed that similar adaptive features are displayed by strains with both ROS^hi^ and ROS^lo^ ROStypes. This result was surprising as we had initially expected to see features in opposition to each other, such that ROS^hi^ strains would have adapted their proteomes to deal with this constant source of oxidative stress while ROS^lo^ strains would perhaps vary in other ways unique to their environment. However, it has been found that other organisms adapt to environments of high oxidative stress by lowering their endogenous ROS levels [67], but conversely, high endogenous ROS can allow for natural adaptive mutations to occur lending to a general increased tolerance to oxidative environments [68]. Thus, the relationship between genetic variation, endogenous ROS, and exogenous ROS is complex, but trends towards similar genetic adaptations as seen in our simulation results. A caveat that should be considered by the reader is that a 5% difference in endogenous ROS generation within our simulations only leads to a nanomolar increase in steady state ROS levels, which may not be substantial enough to warrant changes within the proteome. Furthermore, metadata for the gathered *E. coli* strain sequences was sparse with the only consistent annotation being the strain isolation site, which does not show any correlation to oxidative environments. The analysis of strains and their associated genome sequences could benefit greatly from richer and standardized annotations of observed phenotypes for large-scale studies. In regards to the shared antioxidative features seen in both these ROStypes, proteomics methods to quantify damage sites and create a “proteomic signature” of oxidative stress resistance represents a clear path forward to experimentally verify these predictions in the future [69].

### Dual functionalities of molybdoenzymes potentially contribute to the oxidative damage repair response

The molybdoenzymes within *E. coli* generally catalyze unique redox reactions under aerobic conditions, such as biotin salvage, and also enable the usage of alternative electron acceptors in anaerobic conditions, such as nitrate [56]. Due to their redox capabilities, some molybdoenzymes can also act promiscuously to reverse oxidation events to other metabolites such as methionine [59]. Interestingly, the standard repair system for methionine sulfoxide - specifically in the stressful oxidative environment of the periplasm (MsrPQ) - utilizes molybdenum as its cofactor of choice and is able to reduce both stereoisomers of oxidized methionine [8]. The proposed stability of these molybdoenzymes under oxidative conditions suggests two factors: 1) that these enzymes would be upregulated in response to oxidative stress to reduce their main binding partners that are being oxidized by ROS, and 2) that they may be called upon to carry out their promiscuous repair functions on other oxidized metabolites.

Although the function of molybdoenzymes in anaerobic respiration seems unrelated to oxidative stress, there may be reasons for their use in aerobic conditions. Interestingly, a previous study indicated the metabolite trimethylamine-N-oxide (TMAO) to confer protein structural stability in vitro, by stabilizing charged residues, disordered regions, and preventing protein aggregation [70]. The identified TorZ enzyme in this analysis may then have a role in maintaining reduced TMAO in conditions of oxidative stress. The usage of nitrate as an electron acceptor in anaerobic respiration generates reactive nitrogen species, which, similarly to ROS, damage cysteine and additionally tyrosine residues [71]. Manual analysis of TorZ and BisC structures displayed avoidance of surface-exposed tyrosines, suggesting that similar structural analysis could be carried out for other sites of protein damage.

### Type 1 fimbriae biogenesis operons and their use in oxidative environments

The type 1 fimbriae biogenesis components are interesting to approach from the standpoint of oxidative damage resistance due to three reasons. First, the assembly of the fimbriae depends on an oxidation event to create a single disulfide bond on the components that make up the tip of the fimbriae, which ends up adhering to the environmental surface [72]. Second, there are many similar operons with homologous genes that encode for other fimbriae, supposedly for different environmental conditions [52]. Third, the expression of these components is sensitive to oxygen levels, being inactive under anaerobic conditions [73]. Fourth, recent work has identified this group of proteins as a more discriminatory typing assay to identify UPEC strains [74]. We grouped together ExPEC, AIEC, and APEC strains since their encountered environments are associated with high oxidative stress and because of their similarity in both phylogenetic origin and virulence factors [75–77].

The assembly of the fimbriae begins when a subunit is translocated into the periplasm and bound to a chaperone, which accompanies the subunit to the outer membrane “usher” (collectively known as the chaperone-usher pathway). This binding event depends on the subunit being oxidized by DsbA, a periplasmic enzyme that creates and maintains disulfide bonds [72]. The formation of disulfide bonds would be accelerated by an environment with higher levels of ROS, but would additionally demand the chaperone to have antioxidative properties if the fimbriae were to properly assemble. Additionally, previous studies have shown that most sequence variation on the extracellular fimbriae subunits do not decrease the specificity of adhesion to carbohydrates [78]. However, specific point mutations on the FimH adhesin do provide higher adhesion capabilities [79]. The results presented here suggest that variations are likely implicated in greater stability during translocation and when being presented on the exposed regions of the fimbriae. The existence of the other type 1 fimbriae operons points to a highly adaptable set of adhesins available to an *E. coli* cell. Finally, the finding that the expression of these operons responds to changes in oxygen levels [73] points to a likely link between their antioxidative properties and survival within an oxidative environment.

The two *E. coli* ABU 83972 strains in the cluster of ExPEC/AIEC/APEC strains have been shown to outcompete UPEC strains in colonization of the bladder [80]. The similar properties of their type I fimbriae biogenesis proteins may point towards a similar resistance to oxidative damage in the urinary tract, which is a stressful environment during urinary tract infection [81, 82]. We hypothesize that the greater stability of the biogenesis enzymes under these conditions potentially allow these nonpathogenic strains to assemble their fimbriae and colonize the bladder, outcompeting the UPEC strains. This finding suggests that further experimental work to characterize these relatively unknown fimbriae operon components may elucidate adherence properties of *E. coli* strains.

## Conclusion

In this study, we have applied two protocols in a structural systems biology approach to develop an understanding of the genotype-phenotype relationship. At the systems-level, we utilized strain-specific genome-scale metabolic models to predict levels of endogenous ROS, while also guiding orthologous gene mapping of strains for sequence-level variation analysis. With structural information, we mapped this variation to specific groups of proteins and their location within three-dimensional space. Specific protein groups were identified as differentiating in regards to their antioxidative properties. The analysis of 1764 sequenced strains confers strong evidence pointing towards further experimental study of the contributions of molybdenum-binding enzymes as well as fimbriae assembly proteins in the oxidative stress response. The workflow presented here also demonstrates a method for understanding important features of the structural proteome to enable modeling of different stress responses in silico. Looking forward, the exploration of natural variation in regards to enzymatic capabilities to confer a specific environmental advantage could be applied in the creation of fine-grained metabolic models taking into account the properties of the structural proteome. In the lab, this approach could also guide drug development pipelines by identifying susceptible targets as well as library design for directed evolution experiments in protein engineering.

## Methods

### Construction of the *E. coli* structural proteome

We applied an existing pipeline to create genome-scale models of metabolism with protein structures (GEM-PRO models) [43, 83] for the entire proteome of *E. coli* str. K-12 substr. MG1655 (reference proteome downloaded from UniProt [84] on March 20, 2018), using the current implementation contained in the ssbio Python package [44]. This pipeline results in the selection of a single representative three-dimensional tertiary protein structure, either from experimental data in the Protein Data Bank [85] or from homology models generated from the I-TASSER pipeline [54] and the SWISS-MODEL repository [48]. The representative structure is selected after a number of quality checks, which include 1) aligning the reference sequence to all available structures and ranking them based on their percent identity and sequence completeness (i.e. structures with missing portions outside the N- or C- termini are ranked lower than those with complete inner portions); 2) for experimental structures, ranking based on their experimentally determined resolution; and 3) for homology models, ranking them based on their provided quality scores (c-scores for I-TASSER models [54] and QMEAN scores for SWISS-MODEL models [86]), where I-TASSER models are preferentially chosen over SWISS-MODEL models. The list of available structures is first trimmed based on selected cutoffs for sequence identity and completeness (not missing more than 10% of the length of the sequence on both termini, no insertions or deletions, and over 60% sequence identity to the reference sequence), resolution (< 3 Å), and quality score (QMEAN>-4 or c-score > − 1.5). Next, if experimental structures remain, the one with the highest sequence identity, completeness, and resolution is selected. If there are no experimental structures that remain, the top ranking homology model is selected.

In addition to the methodology reported above, a number of improvements were implemented for this study and are slated for incorporation into the publicly available pipeline in ssbio. These improvements include 1) the selection of representative experimental structures with the consideration of bound substrates or cofactors [87]; 2) the definition of membrane spanning domains in transmembrane proteins by consolidating information as predicted by TMHMM [88] or OPM [89] and as annotated in UniProt; 3) the selection of quaternary structures of protein complexes by a breadth-first search matching algorithm to determine the structure with the highest quality and coverage of annotated subunits, either from biological assemblies in the PDB or from predicted complexes in the SWISS-MODEL repository.

For metal-binding proteins specifically, we implemented a more rigorous selection scheme due to their importance in oxidative stress tolerance. Annotated binding residues were retrieved from each protein’s UniProt entry, and mapped to the correct residue numbering scheme in the structure file. The representative structure for the metal-binding protein was then based on the following four factors: 1) sequence identity, coverage, and resolution as described above; 2) presence of the annotated metal binding site in the solved structure; 3) presence of the metabolic model-annotated metal ion; and 4) presence or absence of any model-annotated cofactors other than the metal ion. The MetalPDB database [38] was used to help expedite this analysis, as they provide extracted metal-binding sites from the PDB structures that can be used to verify the presence of the metal ion along with the residues involved in binding. Per protein, these factors contributed to a weighted score enabling a custom rank-ordering of all available structures, leading to a final structure that best represents the enzyme and its state in the metabolic model.

### Division of the proteome into groups

To delineate search spaces within the structural proteome we created groups of proteins first defined by their localization, and then defined by their functional assignment (Fig. 1a, Additional File 1: Table S1). Localization within a cell is defined by the categories: outer membrane, periplasm, inner membrane, and cytosol. This information was taken from a consensus of a previously generated genome-scale model of proteome synthesis [90], proteomics information from [91], the EchoLOCATION database [92], the UniProt database, and finally predictions from TMHMM [88] if no other information was available. Secondary functional groups were either created with 1) the clusters of orthologous group (COG) categories [93]; 2) metabolic network subsystem as defined in *i*ML1515; 3) metal-binding enzymes as annotated in UniProt; and 4) manual assignment in relation to ROS generation or repair. The manually curated list of proteins (see Additional file 1: Table S2) were collected based on their functional relation to oxidative stress levels in *E. coli*. These include proteins that are known generators of reactive oxygen species, are involved in the repair of oxidative damage to macromolecules, are involved in regulation of metal ion transport, etc. A table summarizing the protein groups can be found in Additional file 1: Table S1.

### Protein property calculations and division into subsequences

The physicochemical properties of each representative protein’s sequence and structure were calculated using a variety of tools and stored as per-residue annotations using ssbio. The properties used in this study include: 1) definitions of protein “sites” (i.e. binding, catalytic, or active sites) as annotated in UniProt, the Catalytic Site Atlas [94], and MetalPDB [38]; 2) regions of protein flexibility and disorder as retrieved from PDBFlex [95] or predicted from sequence with DisEMBL [96] and IUPred [97]; 3) parameters of solvent accessibility as calculated using FreeSASA [98] or predicted using SCRATCH [99]; 4) parameters of residue depth (a calculation of the distance in Angstroms of a residue from the surface of the protein [100]) as calculated using Biopython [101] and MSMS [102]; and 5) definitions of secondary structure using DSSP [103] or predicted using SCRATCH. We additionally incorporated the locations of known oxidizable cysteines from RedoxDB [40], known carbonylatable amino acids (R, K, P, T) from CarbonylDB [39], and predicted disulfide bridges using a distance-based metric (3 Å cutoff) in an extension of the PDB module in Biopython (http://biopython.org/wiki/Struct).

The aforementioned properties were then used to delineate search spaces within single proteins into what we refer to as “protein subsequences” (Fig. 1b). In all cases, there exists at least one of the following methods to compute their definition: 1) “2D subsequences”, which are simply a 3D structural feature predicted from the amino acid sequence (such as running SCRATCH for predicting secondary structure); 2) “2.5D subsequences”, that only apply to sites of a protein that have an annotated site feature in a sequence database such as UniProt and can be mapped to a 3D structure, but for which there exists no structural evidence of the binding (an example of this would be an annotated metal-binding site that is annotated in UniProt, but no metal ion is present in the experimental structure); and finally 3) “3D subsequences” for which clear structural evidence is available (such as a calculated secondary structure).

To give an illustrative example for one subsequence, let us outline the procedure for “surface” residues, If a 3D structure was available for a protein, residue depth and solvent accessibility algorithms are run on this structure. If a 3D structure is not available, we fall back to defining a 2D subsequence by running a solvent accessibility predictor algorithm (SCRATCH) on the protein sequence. Next, a surface residue is defined as one with a relative solvent accessibility of above 25%, and if a 3D structure was available, an additional constraint of residue depth of less than 2.5 Å. These cutoffs were then applied to all residues in a protein sequence and those that meet the cutoff then form a defined surface subsequence (this corresponds to Additional file 1: Table S3, on the first row). Other properties that were utilized to form protein subsequence definitions are: disordered regions, transmembrane domains, solvent-exposed residues surrounding a sphere of a defined radius around a metal-binding site, catalytic site, DNA-binding site, or any other annotated generic site, and residues within ROS-sensitive sites as found in RedoxDB or CarbonylDB. For a summary table describing all cutoffs used, prediction methods, or structural calculations for these features, please see Additional file 1: Table S3.

### Gathering strains, phenotypes, and pathotypes

The proteomes of *E. coli* strains in this study were gathered from three separate sources: 1) the Ecoref strain panel [55]; 2) the *i*ML1515 metabolic network reconstruction resource [42]; and 3) a manually curated set of adherent-invasive *E. coli* (AIEC) strains. The Ecoref strain panel is a published panel of 696 strains that were tested for growth/no growth under a number of media conditions. The sequenced genomes, resulting protein sequences, and pathotype annotations were downloaded from the publicly accessible database (https://evocellnet.github.io/ecoref/download/). Out of the 214 media conditions, 24 of which include a chemical that induces oxidative stress in some manner were selected for this study (see Additional file 1: Table S7). Out of the 696 strains, 676 were selected due to the presence of phenotypic data under the selected media conditions. From the *i*ML1515 resource, the proteomes and pathotype information of 1045 strains of *E. coli* were downloaded from the PATRIC database [104]. The number differs from the original reported number of strains in the resource due to database updates and removal of poorly annotated genomes. The strains obtained from Ecoref and PATRIC also contained pathotype information for 116 of the strains. Finally, the manually curated set of 23 AIEC strains was obtained through literature review and downloading of the individual genomes from various publications [105–111]. Two pathotype groups was created by 1) extra-intestinal pathogenic (ExPEC), adherent-invasive (AIEC), and avian pathogenic (APEC) strains (which have been shown to be similar to ExPEC strains, see [112, 113]) and 2) all other pathotypes. This grouping was chosen to discriminate pathotypes by the oxidative environments they may encounter.

### Simulation of strain-specific endogenous ROS levels

The construction of strain-specific metabolic models follows a previously established protocol [114]. Briefly, this involves creating a presence/absence orthology matrix of proteins in a strain following orthology detection through bidirectional-best BLAST hits (BBH) at an 80% sequence identity cutoff. The orthology matrix is then used to trim reactions in a metabolic model given a protein’s usage in a reaction. Instead of trimming the original metabolic model of *E. coli* K-12 MG1655, the *i*ML1515-ROS model was used as the base model. Based on a previously developed model [41], *i*ML1515-ROS includes 298 reactions that have the potential to produce H_2_O_2_ and O_2_^−^ [42]. Thus, the strain-specific ROS models predicts changes in endogenous ROS production levels based on the deviation from the measured MG1655 ROS production rates.

Ensemble models (sampling different stoichiometric coefficients of the ROS generating reactions) of each strain were then simulated to sample total endogenous production of ROS. Simulations were conducted under glucose minimal media per [41]. The mean H_2_O_2_ and O_2_^−^ production rates were normalized to the growth rate, thus assigning per-strain predictions of mmol H_2_O_2_ gDW^− 1^ and O_2_^−^ gDW^− 1^. The deviations of endogenous ROS production from the “wild-type” MG1655 strain were classified as “high” or “low” if they deviated above or below 5% of the measured ROS production levels, and “non-varying” if otherwise [45]. This cutoff was chosen as it was previously found that increasing endogenous ROS production rates above this level resulted in a higher likelihood of cell death after treatment with antibiotics, indicating that ROS detoxification methods are compromised [41].

### Characterization of strain-specific changes

The orthology matrix used to generate strain-specific models was used to align orthologous strain protein sequences to the K-12 proteins, using default parameters in the Needleman-Wunsch pairwise alignment tool in the EMBOSS package [115]. All orthologous sequences were loaded into their related Protein objects using the ssbio Python package, and alignments were executed in parallel using the Apache Spark Python API (PySpark, https://spark.apache.org). From this, strain-by-feature matrices describing the proteomic features of all strains and the averaged antioxidative properties were generated (see Additional file 1: Table S8, for a description of the types of averaged properties calculated per subsequence). To deal with missing data in the case of portions of protein sequences that have been truncated (either due to technical reasons such as genome sequence or annotation errors, or true deletions of a strain’s sequence compared to MG1655), we only compared sequences where the aligned length was greater than or equal to 80% of the original K-12 sequence length. In the case of proteins absent from certain strains in protein groups, missing values were imputed using mean imputation for percentages of physicochemical properties.

The strain-by-feature matrices are created for principal component analysis (PCA) as follows. For all combinations of protein groups and subsequences, amino acid ratios were calculated relative to the subsequence length. Ratios of certain groupings of amino acids were also calculated, such as from the number of positively charged, bulky, or disorder promoting residues. These groupings were again chosen due to previous hypotheses that enrichment or avoidance of these changes may be associated with resistance to oxidative damage [7, 9, 26, 31, 116]. All groupings are defined in Additional file 1: Table S9.

As an example, let us take the protein group of cytoplasmic, metal-binding enzymes, retrieved from UniProt and defined in row 11 of Additional file 1: Table S1. The subsequences of all of their metal-binding sites are next defined (Additional file 1: Table S3, row 8). Next, important changes within these binding sites are manually defined (Additional file 1: Table S8, rows 12–18). These changes are stored as percentages of the full subsequence length, in order to summarize changes in bulk. If a strain has an overall avoidance of positively charged residues in proximity to the metal-binding site, this is reflected as a lower overall percentage stored for this strain’s protein. Finally, for the group of metal-binding enzymes, the percentages are averaged together and normalized for sequence length. The strain-by-feature matrix thus contains the strain identifiers as the columns, and summarized features of their metal-binding enzymes as the rows.

### Statistical analysis of protein groups

The data set gathered here could potentially be run through unsupervised, semi-supervised, or supervised learning algorithms due to the existence of labels (ROStype or pathotype) for only a portion of the strains. Unsupervised learning was carried out due to the desire to utilize the entire set of observations (strains) along with their features, to understand if variability existed in the dataset without biasing for the assigned labels, which could potentially be incorrect due to their nature: ROStype being solely a simulated prediction and pathotype coming from multiple disparate annotation databases.

For each protein group, subsequence, and associated strains, features were first standardized to be centered around a zero mean with unit variance, using the preprocessing module from the Python package scikit-learn [117]. Features were then filtered for PCA following the hierarchical clustering by rank correlation coefficient method as presented in [26, 118]. Briefly, this clusters the features together based on Spearman rank correlation, and cuts off similar features over a coefficient of 0.9, keeping the feature closest to the center of the cluster as representative. Since the availability of features varied from prediction from sequence and calculation from structure, this allowed for the filtering out of redundant features when both predictions and calculations were available. To further filter down the feature set before PCA, we created manual filters for features specific to a protein group. For example, if the group to inspect was a set of metal-binding proteins, only then would features specific to the metal-binding site (such as the percentage of cysteines in proximity to site) be included in the feature matrix. A table describing these group specific features is included in Additional file 1: Table S4. PCA was then carried out to understand if specific features contributed to the differentiation of the following: 1) growth/no growth phenotypes in different media conditions; 2) strains with predicted ROStypes higher or lower than “wild-type” (K-12) endogenous ROS levels; and 3) annotated strain pathotypes. To do so, observation labels associated with these phenotypes were assigned back to the transformed data. Next, we wanted to identify protein groups which showed the largest separation between phenotypes. Clustering of the transformed data points on the principal components (PC1 and PC2) was carried out using Density-Based Spatial Clustering of Applications with Noise (DBSCAN) [119]. The “performance” of the clustering relative to the labeled phenotypes acted as a proxy to judge which protein groups showed the best clustering. Performance was measured and ranked using the V-measure [49] which reports the harmonic mean of homogeneity (if a cluster contains only one label type) and completeness (if a label type is assigned to the same cluster) and ranges from 0 to 1, with a value of 1 denoting good clustering.

### Training random forest classifiers

To understand if the identified proteome properties can be used to distinguish between ROS^hi^ and ROS^lo^ ROStypes, we trained random forest classifiers using the R package randomForest (version 4.6–14) [120, 121]. Proteomic features were centered and scaled before training. The number of features that were sampled at each split (i.e., the hyperparameter mtry) was tuned using leave-one-out cross-validation by selecting the highest area under the ROC curve that resulted from ten equidistant integers between two and the respective number of features. This cross-validation procedure was implemented in the caret package version 6.0–82 [122].

## Supplementary information

**Additional file 1.**

## Data Availability

The datasets supporting the results of this article are included within the article and the tables within Additional file 1. The methods used to analyze the data are part of a publicly available Python package at https://github.com/SBRG/ssbio. Additional code used to run the analysis is available from the corresponding author on reasonable request.

## Abbreviations

ROS: Reactive oxygen species
GEM-PRO: Genome-scale metabolic model with protein structures
iML1515-ROS: Metabolic model of *E. coli* with additional ROS generating reactions
ROStype: Classification of strain by predicted basal endogenous ROS levels using iML1515-ROS
ROShi: Strain with predicted higher levels (+ 5%) of endogenous ROS relative to K-12 MG1655
ROSlo: Strain with predicted lower levels (− 5%) of endogenous ROS relative to K-12 MG1655
RKPT: Arginine, lysine, proline, threonine - amino acids which are carbonylatable
COG: Clusters of orthologous group
PCA: Principal component analysis
DBSCAN: Density-based spatial clustering of applications with noise
ROC: Receiver operating characteristic curve
AUC: Area under curve in a ROC curve
ExPEC: Extra-intestinal pathogenic *Escherichia coli*
AIEC: Adherent-invasive *Escherichia coli*
APEC: Avian pathogenic *Escherichia coli*
EHEC: Enterohemorrhagic *Escherichia coli*
UPEC: Uropathogenic *Escherichia coli*
TMAO: Trimethylamine-N-oxide

## References

[1] Cabiscol E, Tamarit J, Ros J (2000). Oxidative stress in bacteria and protein damage by reactive oxygen species. Int Microbiol.

[2] Hernández-García D, Wood CD, Castro-Obregón S, Covarrubias L (2010). Reactive oxygen species: a radical role in development?. Free Radic Biol Med.

[3] Naviaux RK (2012). Oxidative shielding or oxidative stress?. J Pharmacol Exp Ther.

[4] Jones RM, Luo L, Ardita CS, Richardson AN, Kwon YM, Mercante JW (2013). Symbiotic lactobacilli stimulate gut epithelial proliferation via Nox-mediated generation of reactive oxygen species. EMBO J.

[5] Rea WJ, Patel KD. Reversibility of chronic disease and hypersensitivity, volume 4: the environmental aspects of chemical sensitivity: CRC Press; 2017. https://market.android.com/details?id=book-pN5CDwAAQBAJ.

[6] Jakob U, Reichmann D (2013). Oxidative stress and redox regulation.

[7] Ghosh K, de Graff AMR, Sawle L, Dill KA (2016). Role of proteome physical chemistry in cell behavior. J Phys Chem B.

[8] Ezraty B, Gennaris A, Barras F, Collet J-F (2017). Oxidative stress, protein damage and repair in bacteria. Nat Rev Microbiol.

[9] Rao RSP, Møller IM (2011). Pattern of occurrence and occupancy of carbonylation sites in proteins. Proteomics..

[10] Maisonneuve E, Ducret A, Khoueiry P, Lignon S, Longhi S, Talla E (2009). Rules governing selective protein carbonylation. PLoS One.

[11] Grimsrud PA, Xie H, Griffin TJ, Bernlohr DA (2008). Oxidative stress and covalent modification of protein with bioactive aldehydes. J Biol Chem.

[12] Vieira-Silva S, Rocha EPC (2008). An assessment of the impacts of molecular oxygen on the evolution of proteomes. Mol Biol Evol.

[13] Oliver CN, Ahn BW, Moerman EJ, Goldstein S, Stadtman ER (1987). Age-related changes in oxidized proteins. J Biol Chem.

[14] Andziak B, O’Connor TP, Qi W, DeWaal EM, Pierce A, Chaudhuri AR (2006). High oxidative damage levels in the longest-living rodent, the naked mole-rat. Aging Cell.

[15] Pérez VI, Buffenstein R, Masamsetti V, Leonard S, Salmon AB, Mele J (2009). Protein stability and resistance to oxidative stress are determinants of longevity in the longest-living rodent, the naked mole-rat. Proc Natl Acad Sci U S A.

[16] Bokov A, Chaudhuri A, Richardson A (2004). The role of oxidative damage and stress in aging. Mech Ageing Dev.

[17] Schindeldecker M, Moosmann B (2015). Protein-borne methionine residues as structural antioxidants in mitochondria. Amino Acids.

[18] Levine RL, Mosoni L, Berlett BS, Stadtman ER (1996). Methionine residues as endogenous antioxidants in proteins. Proc Natl Acad Sci U S A.

[19] Luo S, Levine RL (2009). Methionine in proteins defends against oxidative stress. FASEB J.

[20] St John G, Brot N, Ruan J, Erdjument-Bromage H, Tempst P, Weissbach H (2001). Peptide methionine sulfoxide reductase from Escherichia coli and mycobacterium tuberculosis protects bacteria against oxidative damage from reactive nitrogen intermediates. Proc Natl Acad Sci U S A.

[21] Requejo R, Hurd TR, Costa NJ, Murphy MP (2010). Cysteine residues exposed on protein surfaces are the dominant intramitochondrial thiol and may protect against oxidative damage. FEBS J.

[22] Moosmann B, Behl C (2000). Cytoprotective antioxidant function of tyrosine and tryptophan residues in transmembrane proteins. Eur J Biochem.

[23] Moosmann B (2011). Respiratory chain cysteine and methionine usage indicate a causal role for thiyl radicals in aging. Exp Gerontol.

[24] Krisko A, Radman M (2013). Phenotypic and genetic consequences of protein damage. PLoS Genet.

[25] de Graff AMR, Hazoglou MJ, Dill KA (2016). Highly charged proteins: the Achilles’ heel of aging proteomes. Structure..

[26] Vidovic A, Supek F, Nikolic A, Krisko A (2014). Signatures of conformational stability and oxidation resistance in proteomes of pathogenic bacteria. Cell Rep.

[27] Panda A, Ghosh TC (2014). Prevalent structural disorder carries signature of prokaryotic adaptation to oxic atmosphere. Gene..

[28] Girod M, Enjalbert Q, Brunet C, Antoine R, Lemoine J, Lukac I (2014). Structural basis of protein oxidation resistance: a lysozyme study. PLoS One.

[29] Pieulle L, Magro V, Hatchikian EC (1997). Isolation and analysis of the gene encoding the pyruvate-ferredoxin oxidoreductase of Desulfovibrio africanus, production of the recombinant enzyme in Escherichia coli, and effect of carboxy-terminal deletions on its stability. J Bacteriol.

[30] Lu Z, Imlay JA. The fumarate reductase of Bacteroides thetaiotaomicron, unlike that of Escherichia coli, is configured so that it does not generate reactive oxygen species. MBio. 2017;8. 10.1128/mBio.01873-16. 10.1128/mBio.01873-16 PMC5210497 28049145

[31] Stocker R, Keaney JF (2005). New insights on oxidative stress in the artery wall. J Thromb Haemost.

[32] Valasatava Y, Rosato A, Furnham N, Thornton JM, Andreini C (2018). To what extent do structural changes in catalytic metal sites affect enzyme function?. J Inorg Biochem.

[33] Jang HH, Lee KO, Chi YH, Jung BG, Park SK, Park JH (2004). Two enzymes in one; two yeast peroxiredoxins display oxidative stress-dependent switching from a peroxidase to a molecular chaperone function. Cell..

[34] Ritz D, Lim J, Reynolds CM, Poole LB, Beckwith J (2001). Conversion of a peroxiredoxin into a disulfide reductase by a triplet repeat expansion. Science..

[35] Chabrière E, Charon MH, Volbeda A, Pieulle L, Hatchikian EC, Fontecilla-Camps JC (1999). Crystal structures of the key anaerobic enzyme pyruvate:ferredoxin oxidoreductase, free and in complex with pyruvate. Nat Struct Biol.

[36] Wan B, Zhang Q, Ni J, Li S, Wen D, Li J (2017). Type VI secretion system contributes to Enterohemorrhagic Escherichia coli virulence by secreting catalase against host reactive oxygen species (ROS). PLoS Pathog.

[37] Tsuchiya Y, Peak-Chew SY, Newell C, Miller-Aidoo S, Mangal S, Zhyvoloup A (2017). Protein CoAlation: a redox-regulated protein modification by coenzyme a in mammalian cells. Biochem J.

[38] Andreini C, Cavallaro G, Lorenzini S, Rosato A (2013). MetalPDB: a database of metal sites in biological macromolecular structures. Nucleic Acids Res.

[39] Rao RSP, Zhang N, Xu D, Møller IM. CarbonylDB: a curated data-resource of protein carbonylation sites. Bioinformatics. 2018. 10.1093/bioinformatics/bty123. 10.1093/bioinformatics/bty123 PMC6041944 29509874

[40] Sun M-A, Wang Y, Cheng H, Zhang Q, Ge W, Guo D (2012). RedoxDB—a curated database for experimentally verified protein oxidative modification. Bioinformatics..

[41] Brynildsen MP, Winkler JA, Spina CS, MacDonald IC, Collins JJ (2013). Potentiating antibacterial activity by predictably enhancing endogenous microbial ROS production. Nat Biotechnol.

[42] Monk JM, Lloyd CJ, Brunk E, Mih N, Sastry A, King Z (2017). iML1515, a knowledgebase that computes Escherichia coli traits. Nat Biotechnol.

[43] Brunk E, Mih N, Monk J, Zhang Z, O’Brien EJ, Bliven SE (2016). Systems biology of the structural proteome. BMC Syst Biol.

[44] Mih N, Brunk E, Chen K, Catoiu E, Sastry A, Kavvas E, et al. Ssbio: a python framework for structural systems biology. Bioinformatics. 2018. 10.1093/bioinformatics/bty077. 10.1093/bioinformatics/bty077 PMC6658713 29444205

[45] Imlay JA, Fridovich I (1991). Assay of metabolic superoxide production in Escherichia coli. J Biol Chem.

[46] Baumler DJ, Peplinski RG, Reed JL, Glasner JD, Perna NT (2011). The evolution of metabolic networks of E coli. BMC Syst Biol.

[47] Monk JM, Charusanti P, Aziz RK, Lerman JA, Premyodhin N, Orth JD (2013). Genome-scale metabolic reconstructions of multiple Escherichia coli strains highlight strain-specific adaptations to nutritional environments. Proc Natl Acad Sci U S A.

[48] Biasini M, Bienert S, Waterhouse A, Arnold K, Studer G, Schmidt T (2014). SWISS-MODEL: modelling protein tertiary and quaternary structure using evolutionary information. Nucleic Acids Res.

[49] Rosenberg A, Hirschberg J (2007). V-measure: a conditional entropy-based external cluster evaluation measure. Proceedings of the 2007 joint conference on empirical methods in natural language processing and computational natural language learning (EMNLP-CoNLL).

[50] Conover MS, Hadjifrangiskou M, Palermo JJ, Hibbing ME, Dodson KW, Hultgren SJ (2016). Metabolic requirements of Escherichia coli in intracellular bacterial communities during urinary tract infection pathogenesis. MBio..

[51] Hull R, Rudy D, Donovan W, Svanborg C, Wieser I, Stewart C (2000). Urinary tract infection prophylaxis using Escherichia coli 83972 in spinal cord injured patients. J Urol.

[52] Korea C-G, Badouraly R, Prevost M-C, Ghigo J-M, Beloin C (2010). Escherichia coli K-12 possesses multiple cryptic but functional chaperone-usher fimbriae with distinct surface specificities. Environ Microbiol.

[53] Puorger C, Vetsch M, Wider G, Glockshuber R (2011). Structure, folding and stability of FimA, the main structural subunit of type 1 pili from uropathogenic Escherichia coli strains. J Mol Biol.

[54] Roy A, Kucukural A, Zhang Y (2010). I-TASSER: a unified platform for automated protein structure and function prediction. Nat Protoc.

[55] Galardini M, Koumoutsi A, Herrera-Dominguez L, Cordero Varela JA, Telzerow A, Wagih O, et al. Phenotype inference in anEscherichia colistrain panel. Elife. 2017;6. 10.7554/eLife.31035. 10.7554/eLife.31035 PMC5745082 29280730

[56] Iobbi-Nivol C, Leimkühler S (1827). Molybdenum enzymes, their maturation and molybdenum cofactor biosynthesis in Escherichia coli. Biochim Biophys Acta.

[57] Hille R (1996). The mononuclear molybdenum enzymes. Chem Rev.

[58] Fischer M, Thöny B, Leimkühler S (2010). 7.17 - the biosynthesis of folate and Pterins and their enzymology. Comprehensive natural products II.

[59] Ezraty B, Bos J, Barras F, Aussel L (2005). Methionine sulfoxide reduction and assimilation in Escherichia coli: new role for the biotin sulfoxide reductase BisC. J Bacteriol.

[60] Ansaldi M, Théraulaz L, Baraquet C, Panis G, Méjean V (2007). Aerobic TMAO respiration in Escherichia coli. Mol Microbiol.

[61] Méjean V, Iobbi-Nivol C, Lepelletier M, Giordano G, Chippaux M, Pascal MC (1994). TMAO anaerobic respiration in Escherichia coli: involvement of the tor operon. Mol Microbiol.

[62] del Campillo CA, Campbell A (1996). Alternative gene for biotin sulfoxide reduction inEscherichia coli K-12. J Mol Evol.

[63] Xi H, Schneider BL, Reitzer L (2000). Purine catabolism in Escherichia coli and function of xanthine dehydrogenase in purine salvage. J Bacteriol.

[64] Belenky P, Ye JD, Porter CBM, Cohen NR, Lobritz MA, Ferrante T (2015). Bactericidal antibiotics induce toxic metabolic perturbations that Lead to cellular damage. Cell Rep.

[65] Cookson AL, Cooley WA, Woodward MJ (2002). The role of type 1 and curli fimbriae of Shiga toxin-producing Escherichia coli in adherence to abiotic surfaces. Int J Med Microbiol.

[66] Shaikh N, Holt NJ, Johnson JR, Tarr PI (2007). Fim operon variation in the emergence of Enterohemorrhagic Escherichia coli: an evolutionary and functional analysis. FEMS Microbiol Lett.

[67] Smith SW, Latta LC, Denver DR, Estes S (2014). Endogenous ROS levels in C elegans under exogenous stress support revision of oxidative stress theory of life-history tradeoffs. BMC Evol Biol.

[68] Aubron C, Glodt J, Matar C, Huet O, Borderie D, Dobrindt U (2012). Variation in endogenous oxidative stress in Escherichia coli natural isolates during growth in urine. BMC Microbiol.

[69] McDonagh B (2017). Detection of ROS induced proteomic signatures by mass spectrometry. Front Physiol.

[70] Levine ZA, Larini L, LaPointe NE, Feinstein SC, Shea J-E (2015). Regulation and aggregation of intrinsically disordered peptides. Proc Natl Acad Sci U S A.

[71] Souza JM, Chen Q, Blanchard-Fillion B, Lorch SA, Hertkorn C, Lightfoot R, Dansette PM, Snyder R, Delaforge M, Gibson GG, Greim H, Jollow DJ (2001). Reactive nitrogen species and proteins: biological significance and clinical relevance. Biological reactive intermediates VI: chemical and biological mechanisms in susceptibility to and prevention of environmental diseases.

[72] Crespo MD, Puorger C, Schärer MA, Eidam O, Grütter MG, Capitani G (2012). Quality control of disulfide bond formation in pilus subunits by the chaperone FimC. Nat Chem Biol.

[73] Floyd KA, Moore JL, Eberly AR, Good JAD, Shaffer CL, Zaver H (2015). Adhesive fiber stratification in uropathogenic Escherichia coli biofilms unveils oxygen-mediated control of type 1 pili. PLoS Pathog.

[74] Ren Y, Palusiak A, Wang W, Wang Y, Li X, Wei H (2016). A high-resolution typing assay for Uropathogenic Escherichia coli based on Fimbrial diversity. Front Microbiol.

[75] Palmela C, Chevarin C, Xu Z, Torres J, Sevrin G, Hirten R, et al. Adherent-invasive Escherichia coli in inflammatory bowel disease. Gut. 2017. 10.1136/gutjnl-2017-314903. 10.1136/gutjnl-2017-314903 29141957

[76] Bringer M-A, Glasser A-L, Tung C-H, Méresse S, Darfeuille-Michaud A (2006). The Crohn’s disease-associated adherent-invasive Escherichia coli strain LF82 replicates in mature phagolysosomes within J774 macrophages. Cell Microbiol.

[77] Martinez-Medina M, Mora A, Blanco M, López C, Alonso MP, Bonacorsi S (2009). Similarity and divergence among adherent-invasive Escherichia coli and extraintestinal pathogenic E. coli strains. J Clin Microbiol.

[78] Bouckaert J, Mackenzie J, de Paz JL, Chipwaza B, Choudhury D, Zavialov A (2006). The affinity of the FimH fimbrial adhesin is receptor-driven and quasi-independent of Escherichia coli pathotypes. Mol Microbiol.

[79] Dreux N, Denizot J, Martinez-Medina M, Mellmann A, Billig M, Kisiela D (2013). Point mutations in FimH adhesin of Crohn’s disease-associated adherent-invasive Escherichia coli enhance intestinal inflammatory response. PLoS Pathog.

[80] Roos V, Ulett GC, Schembri MA, Klemm P (2006). The asymptomatic bacteriuria Escherichia coli strain 83972 outcompetes uropathogenic E. coli strains in human urine. Infect Immun.

[81] Kurutas EB, Ciragil P, Gul M, Kilinc M (2005). The effects of oxidative stress in urinary tract infection. Mediators Inflamm.

[82] Hryckowian AJ, Welch RA (2013). RpoS contributes to phagocyte oxidase-mediated stress resistance during urinary tract infection by Escherichia coli CFT073. MBio..

[83] Chang RL, Andrews K, Kim D, Li Z, Godzik A, Palsson BO (2013). Structural systems biology evaluation of metabolic thermotolerance in Escherichia coli. Science..

[84] The UniProt Consortium (2017). UniProt: the universal protein knowledgebase. Nucleic Acids Res.

[85] Berman HM (2000). The protein data Bank. Nucleic Acids Res.

[86] Benkert P, Biasini M, Schwede T (2011). Toward the estimation of the absolute quality of individual protein structure models. Bioinformatics..

[87] Yang L, Mih N, Yurkovich JT, Park JH, Seo S, Kim D, et al. Multi-scale model of the proteomic and metabolic consequences of reactive oxygen species. bioRxiv. 2017:227892. 10.1101/227892.

[88] Krogh A, Larsson B, von Heijne G, Sonnhammer EL (2001). Predicting transmembrane protein topology with a hidden Markov model: application to complete genomes. J Mol Biol.

[89] Lomize MA, Lomize AL, Pogozheva ID, Mosberg HI (2006). OPM: orientations of proteins in membranes database. Bioinformatics..

[90] Liu JK, O’Brien EJ, Lerman JA, Zengler K, Palsson BO, Feist AM (2014). Reconstruction and modeling protein translocation and compartmentalization in Escherichia coli at the genome-scale. BMC Syst Biol.

[91] Schmidt A, Kochanowski K, Vedelaar S, Ahrné E, Volkmer B, Callipo L (2016). The quantitative and condition-dependent Escherichia coli proteome. Nat Biotechnol.

[92] Horler RSP, Butcher A, Papangelopoulos N, Ashton PD, Thomas GH (2009). EchoLOCATION: an in silico analysis of the subcellular locations of Escherichia coli proteins and comparison with experimentally derived locations. Bioinformatics..

[93] Galperin MY, Makarova KS, Wolf YI, Koonin EV (2015). Expanded microbial genome coverage and improved protein family annotation in the COG database. Nucleic Acids Res.

[94] Porter CT, Bartlett GJ, Thornton JM (2004). The catalytic site atlas: a resource of catalytic sites and residues identified in enzymes using structural data. Nucleic Acids Res.

[95] Hrabe T, Li Z, Sedova M, Rotkiewicz P, Jaroszewski L, Godzik A (2016). PDBFlex: exploring flexibility in protein structures. Nucleic Acids Res.

[96] Linding R, Jensen LJ, Diella F, Bork P, Gibson TJ, Russell RB (2003). Protein disorder prediction: implications for structural proteomics. Structure..

[97] Dosztányi Z, Csizmok V, Tompa P, Simon I (2005). IUPred: web server for the prediction of intrinsically unstructured regions of proteins based on estimated energy content. Bioinformatics..

[98] Mitternacht S (2016). FreeSASA: an open source C library for solvent accessible surface area calculations. F1000Res.

[99] Cheng J, Randall AZ, Sweredoski MJ, Baldi P (2005). SCRATCH: a protein structure and structural feature prediction server. Nucleic Acids Res.

[100] Chakravarty S, Varadarajan R (1999). Residue depth: a novel parameter for the analysis of protein structure and stability. Structure..

[101] Hamelryck T, Manderick B (2003). PDB file parser and structure class implemented in python. Bioinformatics..

[102] Sanner MF, Olson AJ, Spehner J-C (1996). Reduced surface: an efficient way to compute molecular surfaces. Biopolymers..

[103] Frishman D, Argos P (1995). Knowledge-based protein secondary structure assignment. Proteins..

[104] Antonopoulos DA, Assaf R, Aziz RK, Brettin T, Bun C, Conrad N, et al. PATRIC as a unique resource for studying antimicrobial resistance. Brief Bioinform. 2017. 10.1093/bib/bbx083. 10.1093/bib/bbx083 PMC6781570 28968762

[105] Desilets M, Deng X, Deng X, Rao C, Ensminger AW, Krause DO (2016). Genome-based definition of an inflammatory bowel disease-associated adherent-invasive Escherichia coli Pathovar. Inflamm Bowel Dis.

[106] O’Brien CL, Bringer M-A, Holt KE, Gordon DM, Dubois AL, Barnich N, et al. Comparative genomics of Crohn’s disease-associated adherent-invasive Escherichia coli. Gut. 2016. 10.1136/gutjnl-2015-311059. 10.1136/gutjnl-2015-311059 27196580

[107] Krause DO, Little AC, Dowd SE, Bernstein CN (2011). Complete genome sequence of adherent invasive Escherichia coli UM146 isolated from Ileal Crohn’s disease biopsy tissue. J Bacteriol.

[108] Clarke DJ, Chaudhuri RR, Martin HM, Campbell BJ, Rhodes JM, Constantinidou C (2011). Complete genome sequence of the Crohn’s disease-associated adherent-invasive Escherichia coli strain HM605. J Bacteriol.

[109] Nash JH, Villegas A, Kropinski AM, Aguilar-Valenzuela R, Konczy P, Mascarenhas M (2010). Genome sequence of adherent-invasive Escherichia coli and comparative genomic analysis with other E coli pathotypes. BMC Genomics.

[110] Zhang Y, Rowehl L, Krumsiek JM, Orner EP, Shaikh N, Tarr PI (2015). Identification of candidate adherent-invasive E coli signature transcripts by genomic/transcriptomic analysis. PLoS One.

[111] Dogan B, Suzuki H, Herlekar D, Sartor RB, Campbell BJ, Roberts CL (2014). Inflammation-associated adherent-invasive Escherichia coli are enriched in pathways for use of propanediol and iron and M-cell translocation. Inflamm Bowel Dis.

[112] Moulin-Schouleur M, Répérant M, Laurent S, Brée A, Mignon-Grasteau S, Germon P (2007). Extraintestinal pathogenic Escherichia coli strains of avian and human origin: link between phylogenetic relationships and common virulence patterns. J Clin Microbiol.

[113] Ron EZ (2006). Host specificity of septicemic Escherichia coli: human and avian pathogens. Curr Opin Microbiol.

[114] Monk J, Bosi E, Fondi M (2018). Integration of comparative genomics with genome-scale metabolic modeling to investigate strain-specific phenotypical differences. Metabolic network reconstruction and modeling: methods and protocols.

[115] Needleman SB, Wunsch CD (1970). A general method applicable to the search for similarities in the amino acid sequence of two proteins. J Mol Biol.

[116] Kitazoe Y, Kishino H, Hasegawa M, Nakajima N, Thorne JL, Tanaka M (2008). Adaptive threonine increase in transmembrane regions of mitochondrial proteins in higher primates. PLoS One.

[117] Pedregosa F, Varoquaux G, Gramfort A, Michel V, Thirion B, Grisel O (2011). Scikit-learn: machine learning in python. J Mach Learn Res.

[118] Smole Z, Nikolic N, Supek F, Šmuc T, Sbalzarini IF, Krisko A (2011). Proteome sequence features carry signatures of the environmental niche of prokaryotes. BMC Evol Biol.

[119] Ester M, Kriegel H-P, Sander J, Xu X (1996). A density-based algorithm for discovering clusters in large spatial databases with noise. Kdd.

[120] Breiman L (2001). Random forests. Mach Learn.

[121] Team RC (2013). A language and environment for statistical computing.

[122] Kuhn M (2008). Others. Building predictive models in R using the caret package. J Stat Softw.

